# The relationship between resource abundance and insect herbivory on islands

**DOI:** 10.1371/journal.pone.0256183

**Published:** 2021-08-16

**Authors:** Bora Shin, Jae-Young Lee, Nang-Hee Kim, Sei-Woong Choi

**Affiliations:** 1 Department of Biology, Mokpo National University, Muan, Jeonnam, Korea; 2 Department of Environmental Education, Mokpo National University, Muan, Jeonnam, Korea; 3 National Institute of Ecology, Seocheon, Chungnam, Korea; Inha University, REPUBLIC OF KOREA

## Abstract

We examined the relationship between resource abundance and the feeding activity of phytophagous insects on three common island plants. The aim was to investigate the correlation between phytophagous insects’ abundance and availability of food and island geography. We collected 30,835 leaves from three tree species groups (*Mallotus japonicus*, *Prunus* species, and *Quercus* species) on 18 islands in southwest Korea. The number of plant resources for herbivores varied: the number of leaves per shoot was the highest in *Mallotus*, leaf weight and the water content per leaf was significantly lower in *Quercus* species. External feeding was higher for *Prunus* and *Quercus* species, whereas the internal feeding type was significantly higher for *Quercus* species. Geography (area and distance), elevation and food resource (elevation, number of plant species, and the forest cover rate) had a variable effect on phytophagous insects feeding activities: distance and the number of plant species were more explainable to the external feeding guild. In contrast, area and forest cover were more to the internal feeding guild.

## Introduction

An island is an isolated landmass surrounded by water and typically comprised of diverse habitats from seashores to forested areas despite their limited size. Islands offer an important opportunity to investigate the evolution theory because island biota often evolved peculiar characteristics to adapt to the island environment over time. MacArthur & Wilson [1] suggested an equilibrium theory of island biogeography that predicts that the number of species on an island is determined by island size and isolation. This theory postulated that species inhabiting islands closer to the mainland are more likely to immigrate than those further from the mainland. It also proposed that species living on small islands have a higher probability of going extinct than those on larger islands due to competition [1]. The island biogeography theory is now widely accepted as an established ecological theory. Multiple studies show a strong relationship between the number of species on an island and the island’s area [1–14]. Also, Lack [15] suggested that island species diversity is closely related to habitat diversity with more distant islands having lower diversity caused by low habitat heterogeneity due to impoverishment [16].

About 925,000 insects comprise more than half of the living organisms on Earth and show great morphological and functional diversity [17]. Insects can be divided into three functional groups based on their feeding strategies: phytophagous, predacious, and saprophagous. Phytophagous insects, the focus of this study consume plant materials and comprise a quarter of the total insect species. They include more than nine orders: Coleoptera, Collembola, Diptera, Hemiptera, Hymenoptera, Lepidoptera, Orthoptera, Phasmida, and Thysanoptera. Phytophagous insects are an important link between plants and secondary consumers, including predaceous insects, birds, bats, and mammals [18]. Leaf damage, produced by the feeding activities of phytophagous insects, traces the typical interaction between plants and animals. Phytophagous insects can be further divided into two categories based on their feeding mechanisms and leaf damage: external feeder (leaf chewer) regarded as generalist [19], and internal feeder (gall-maker, leaf miner, and sap-sucker) mostly specialists [20].

Here, we measured leaf damage in three plant species to investigate the food plants abundance for phytophagous insects in islands of different sizes and distances from the mainland. The resource abundance hypothesis [21] predicts that plants that offer more resources can support more species and greater abundances of insect herbivores [22]. Since plants and insects arrive and colonize islands independently, phytophagous insects, especially on remote islands, might face the unfavorable condition of lacking their preferred host plant [23]. This mismatching of plants and herbivores can cause insects to either fail to survive or obligatorily subsist on less preferred plant species, causing their larvae to develop more slowly and in reduced numbers. The resulting reduction in herbivore pressure could lead plants to evolve relaxed defenses and alter their chemical traits [24]. Thus, we also measured the number of food resources and leaf traits in the three plant groups to investigate their effect on herbivore survival.

Studies on herbivore-caused leaf damage on island plants are rare [25, 26]. The abundance of host plant individuals or plant biomass is the main resource affecting herbivores’ distribution and population oscillation [22, 27]. We investigated the feeding activities of phytophagous insect species on three plant species on the surveyed islands to determine whether abundant plant resources are beneficial for the abundance of phytophagous insects. We also tested the island biogeography theory using island characteristics, including island size and distance from the mainland, on the abundance of phytophagous insects. We hypothesized that leaf damage would be closely related to island area size and distance from the mainland as postulated by the island biogeography theory since phytophagous insects’ diversity and abundance are closely related [28]. The abundance of phytophagous insects on the island measured as leaf damage was related to food abundance [1].

## Materials and methods

### Study area

**Fig 1:**
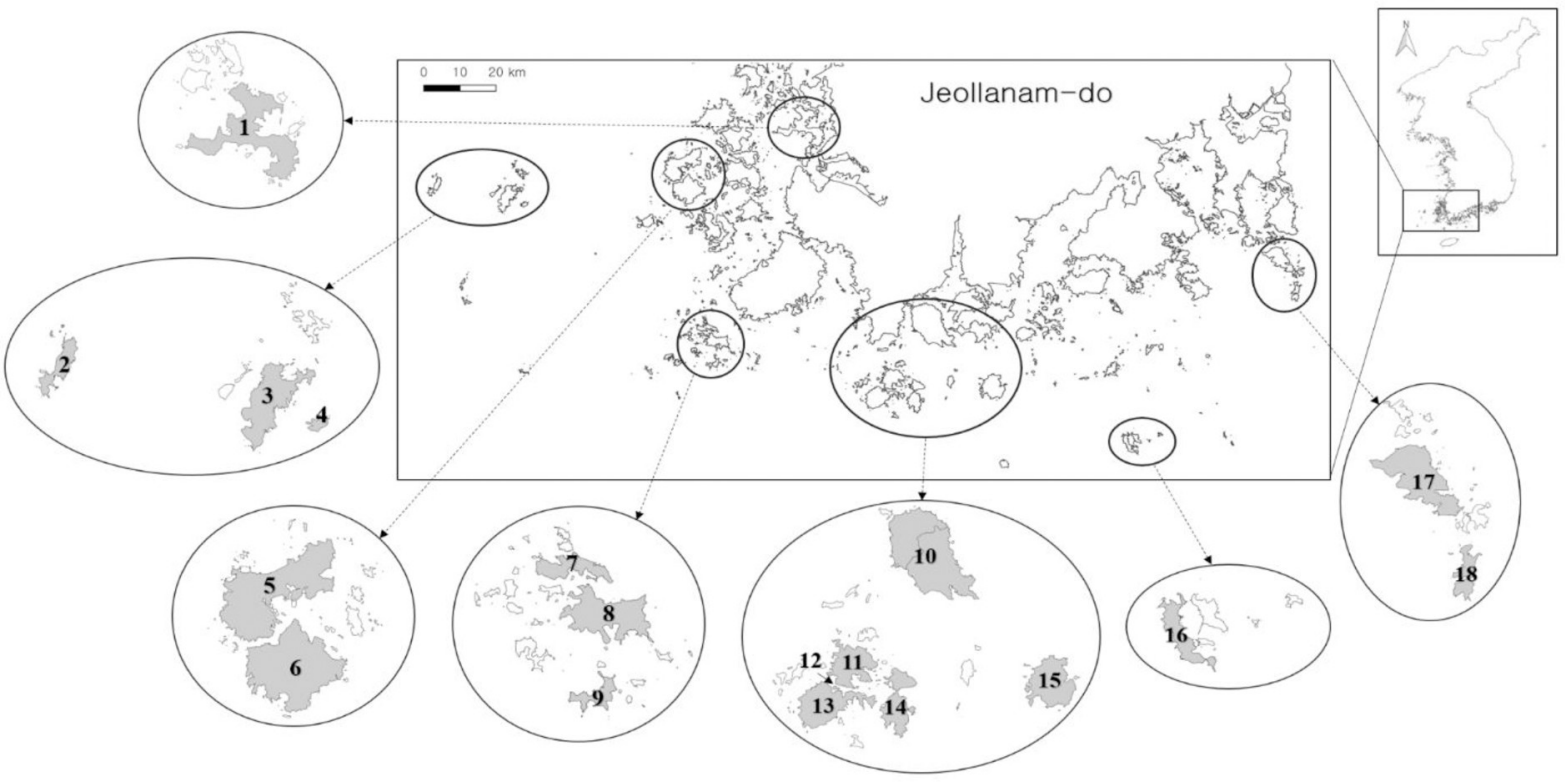
**Map showing the surveyed 18 islands in southwest Korea:** 1. Aphaedo, 2. Hongdo, 3. Heuksando, 4. Yongsando, 5. Bigeumdo, 6. Dochodo, 7. Sangjodo, 8. Hajodo, 9. Gwanmaedo, 10. Wando, 11. Nohwado, 12. Jangsado, 13. Bogildo, 14. Soando, 15. Cheongsando, 16. Geomundo (Seodo), 17. Geumodo, 18. Yeondo.

Korea has about 3,348 islands, of which 2,878 are uninhabited. About 60% of the islands are located in the southwest (Jeollanam-do province), and many are part of the Dadohaehaesang Maritime National Park (Fig **1**). We surveyed the leaves of three plant species groups on 18 islands in the national Park. Each island’s area and maximum elevation were obtained from Korea’s public data portal [29], and the shortest distance from the mainland was measured using Google Earth (https://earth.google.com). The flora on each island and the number of plant species were obtained from the National Park Service [30] (Table 1). Forest cover area (m^2^) for the surveyed island was acquired by processing the vector data from the land cover map from the Ministry of Environment, Korea (http://egis.me.go.kr/main.do). The data comprised seven land cover classes: urban, agricultural, forest (deciduous, coniferous, and mixed), grasses, wetland, bare ground, and water. Images were processed using Geographical Information Systems QGis version 2.18.18 (https://www.qgis.org/en/site/index.html) at a 1:25,000 scale. We used the proportion of forest area (%) as the forest cover of the island.

**Table 1:** Geographic data and the number of examined leaves of three common plant species on 18 surveyed islands in southwest Korea.

Island	Area (km^2^)	Distance from mainland (km)	Maximum elevation (m)	No. of plant species on island	Forest cover (%)	Examined number of leaves		
						*Mallotus japonicus*	*Prunus spp*.	*Quercus spp*.
Aphaedo	48.84	10.8	234	451	25.51	786	437	562
Bigeumdo	48.06	41.5	220	225	26.9	742	485	646
Bogildo	32.14	19	433	407	86.22	767	397	444
Cheongsando	32.96	32.6	385	264	66.71	763	570	449
Dochodo	44.04	40.3	230	251	29.09	767	378	437
Geomundo (Seodo)	7.21	47.4	237	248	64.37	824	595	431
Geumodo	27.5	18.8	382	395	72.84	807	534	401
Gwanmaedo	4	46.4	219	253	82.34	770	499	466
Hajodo	17	44.9	234	330	71.16	755	521	460
Heuksando	21.7	90	345	522	93.65	730	446	432
Jangsado	0.28	17.2	65	79	89.93	843	485	445
Nohwado	25.3	13.6	148	584	47.45	744	389	375
Sangjodo	6.87	47.3	221	218	67.78	718	457	363
Soando	23.22	21.3	350	437	58.49	795	424	453
Wando	90.07	9.9	644	105	63.03	784	407	604
Yeondo	6.93	28	231	348	58.01	783	428	454
Yeongsando	1.91	85.5	165	359	92.84	933	479	356
Hongdo	6.47	115	368	330	82.28	784	592	439

### Sampling method

We sampled leaves from three groups of common island plant species: *Mallotus japonicus* (Euphorbiaceae, MAL); *Prunus yedoensis*, *P*. *takesimensis*, and *P*. *jamasakura* (Rosaceae); and *Quercus dentata*, *Q*. *acutissima*, and *Q*. *serrata* (Fagaceae). We sampled leaves during June and July in two years (2017 and 2018), when the leaves are full-grown, and the feeding activity of phytophagous insects was the highest, while the leaf damage by aging was relatively small [31]. Since *Prunus* and *Quercus* species distribution differed on each island, we grouped these plant species into a *Prunus* group (PRU) and a *Quercus* group (QUE). We randomly sampled ten current-year shoots from nine individual trees of each species and counted and measured external and internal leaf damage observed by the eye. To avoid counting errors, one author (BS) consistently counted the leaf damage.

We classified leaf damage caused by phytophagous insects into chewers (external leaf damage) and gallers or miners (internal leaf damage). Leaf damage by chewers was coded into six grades based on the damage proportion of each leaf: 0 = no damage; 1 = 1~10% damage; 2 = 11~25%; 3 = 26~50%; 4 = 51~75%; and 5 = 76~100% [25, 32, 33]. To calculate the chewing rate of each plant species per island, we first weighted each leaf damage grade differently to a fixed value: 1–1, 2–11, 3–26, 4–51, and 5–76, then multiplied this fixed value with the damaged number leaves for each grade per plant. The chewing rate index of each plant species per island was obtained by dividing the summation of the weighted value by the examined leaves of each island.

Leaf damage by gallers and miners was counted via galls and leaf mines on each leaf. The internal feeding value was obtained by summing the numbers of galls and mines of each island. Leaf damages by galls and leaf mines were relatively scarce compared to chewers. Thus, the internal feeding value was obtained by multiplying ten after averaging the summing numbers of galls and mines of each island.

All field work was conducted with the permission of the Korea National Park Service.

### Leaf traits (LMA, water content)

We collected ten leaves randomly from each tree to measure water content and leaf mass per area (LMA). We weighted a group of these ten leaves. We made a disk (6 mm diameter) per leaf using a puncher, totaling a group of ten disks per plant species, and then dried these ten disks for 48 hrs at 60°C in a drying oven. We measured the weight of each group of ten disks before and after drying. LMA and water content were calculated with the following formula [32, 34].


$$\mathrm{LMA}({\mathrm{mg}∙\mathrm{mm}}^{‐2})=W_{\mathrm{dry}}/A_{\mathrm{area}}$$

$$\mathrm{Water}\;\mathrm{content}\;(\%)=\frac{W_{\mathrm{fresh}}‐W_{\mathrm{dry}}}{W_{\mathrm{fresh}}}$$

$$W_{\mathrm{fresh}}\;:\;\mathrm{Weight}\;\mathrm{of}\;\mathrm{fresh}\;\mathrm{leaf}$$

$$W_{\mathrm{dry}}\;:\;\mathrm{Weight}\;\mathrm{of}\;\mathrm{dried}\;\mathrm{leaf}$$

$$A_{\mathrm{area}}\;:\;\mathrm{Area}\;\mathrm{of}\;\mathrm{leaf}\;\mathrm{disk}$$

### Analysis

We tested the effect of the sampling procedure on phytophagous herbivory based on fixed effects in hierarchical sampling using the island, tree species, numbers of individual trees and shoot sampled, and year and week of sampling date as random effects. We modeled external and internal feeding damage with geography (each islands’ area and distance from the mainland) and habitat diversity expressed as maximum elevation and food resources (number of plant species recorded and the forest cover) using generalized linear models (GLMs). We log-transformed area and distance to reduce skew. We built GLMs with external and internal feeding damage rates as response variables, two sets of explanatory variables: geography (the island area and distance), habitat diversity, and plant resources (plant species richness and the forest cover). In these models, we calculated the independent contribution (R^2^) of each predictor variable and the significance level at 0.05% after 999 randomizations using the “hier.part package” in R. All analyses were carried out in R (R Core Team, 2018).

## Results

### Resource abundance and insect herbivory

**Fig 2:**
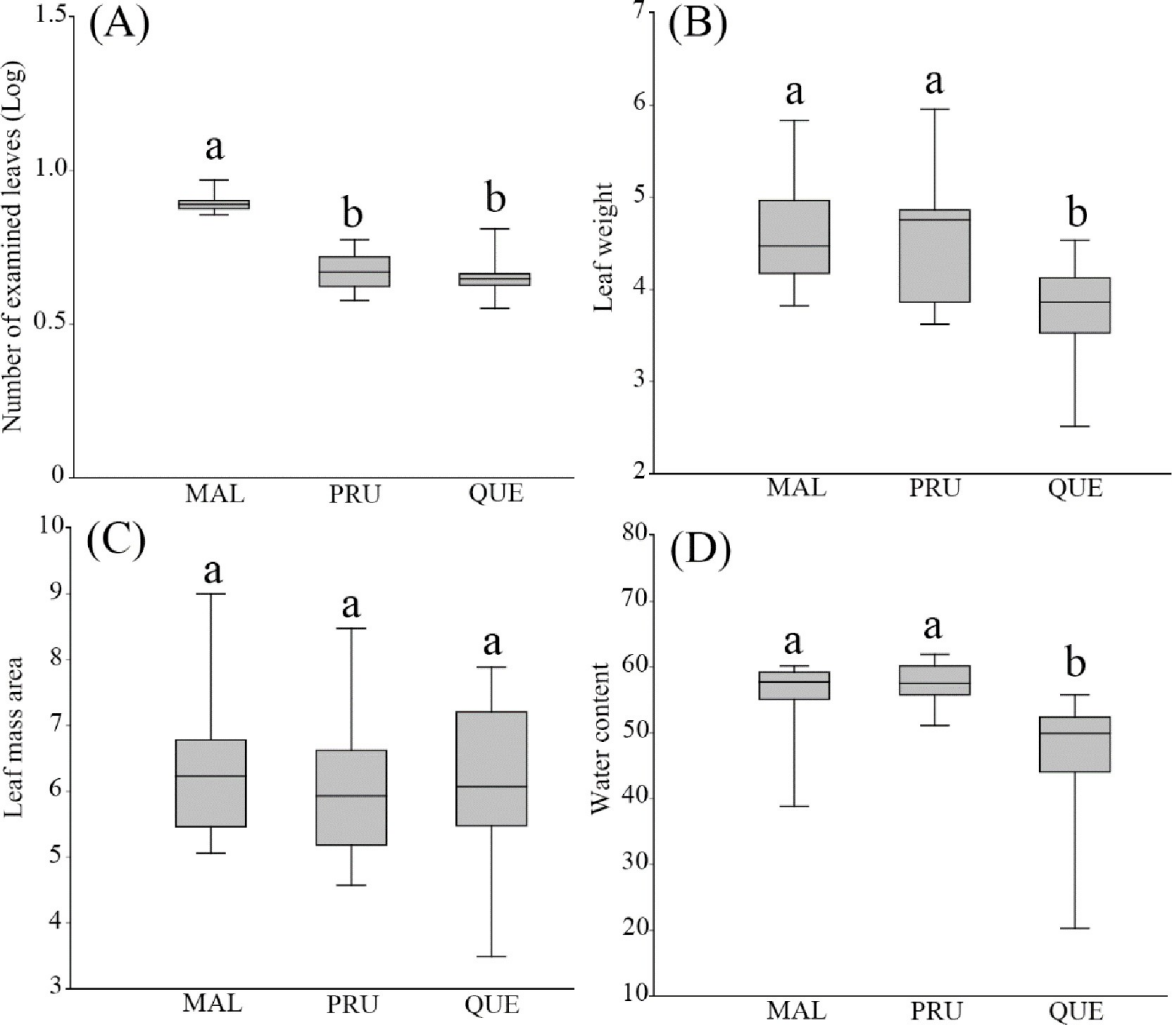
**Box plot of the number of examined leaves (A), the fresh leaf weight (B), LMA (leaf mass per area) (C), and water content (D) for the three plant species across 18 islands.** Different alphabet above the bar indicates the significant difference at *P* < 0.05.

A total of 30,835 leaves from 610 individual trees on 18 islands were collected. The MAL leaves were the most abundant, with 14,095 leaves (45.7%), followed by PRU (8,523 leaves, 27.6%) and QUE (7,655 leaves, 24.8%). There was also a significant difference in the total leaves from the plants when we randomly sampled ten current-year shoots from nine individuals of each plant species (ANOVA F_2,51_ = 141.8, *P* < 0.001, Fig 2A).

The sampling effect to examine the phytophagous insects on three plant species on 18 islands were negligible: the number of individual tree effect was the highest in the hierarchical sampling (10.38%), and the variances of the island, tree species, the number of shoots sampled, year, and week were 3.74, 5.99, 3.25, 3.25, 3.25%, respectively. There was no difference between the model using individual trees as a fixed effect and or not (Likelihood ratio 1.66, *P* = 0.20).

We investigated the fresh leaf weight, LMA, and water content for the three plant species (MAL n = 162, PRU n = 159, QUE n = 162). The average of the fresh leaf weight varied: 41.35 (±1.18 s.e.) mg (MAL), 39.61 (±1.50) mg (PRU), and 33.81 (±1.14) mg (QUE). QUE was significantly lighter than the other plant species (ANOVA F_2,51_ = 9.49, *P* < 0.001, Fig 2B). The average LMA did not differ: PRU 6.01 (±0.25 s.e.) mg.mm^−2^, MAL 6.32 (±0.26) mg.mm^−2^, QUE 6.20 (±0.27) mg.mm^−2^, and was not significantly different (F_2,51_ = 0.37, *P* = 0.69, Fig 2C). The average water content also varied: 56.3 (±1.18 s.e.)% (MAL), 57.6 (±0.65)% (PRU), and 47.2 (±1.90)% (QUE). The water content was significantly lower in QUE (F_2,51_ = 17.72, *P* < 0.001, Fig 2D).

### Insect herbivory

Chewers were the most active phytophagous insects, damaging 23,695 leaves (76.8% of the total leaves): 80.8% (MAL), 79.6% (PRU), and 67.1% (QUE). We found no significant correlation between herbivory rate and the examined leaves of three plant species (Chewers: MAL Pearson r = −0.06, *P* = 0.80; PRU r = −0.31, *P* = 0.22, QUE r = −0.26, *P* = 0.31; gallers and miners: MAL Pearson r = 0.15, *P* = 0.55; QUE r = −0.26, *P* = 0.31), except the internal feeding of PRU (r = 0.57, *P* < 0.05).

**Fig 3:**
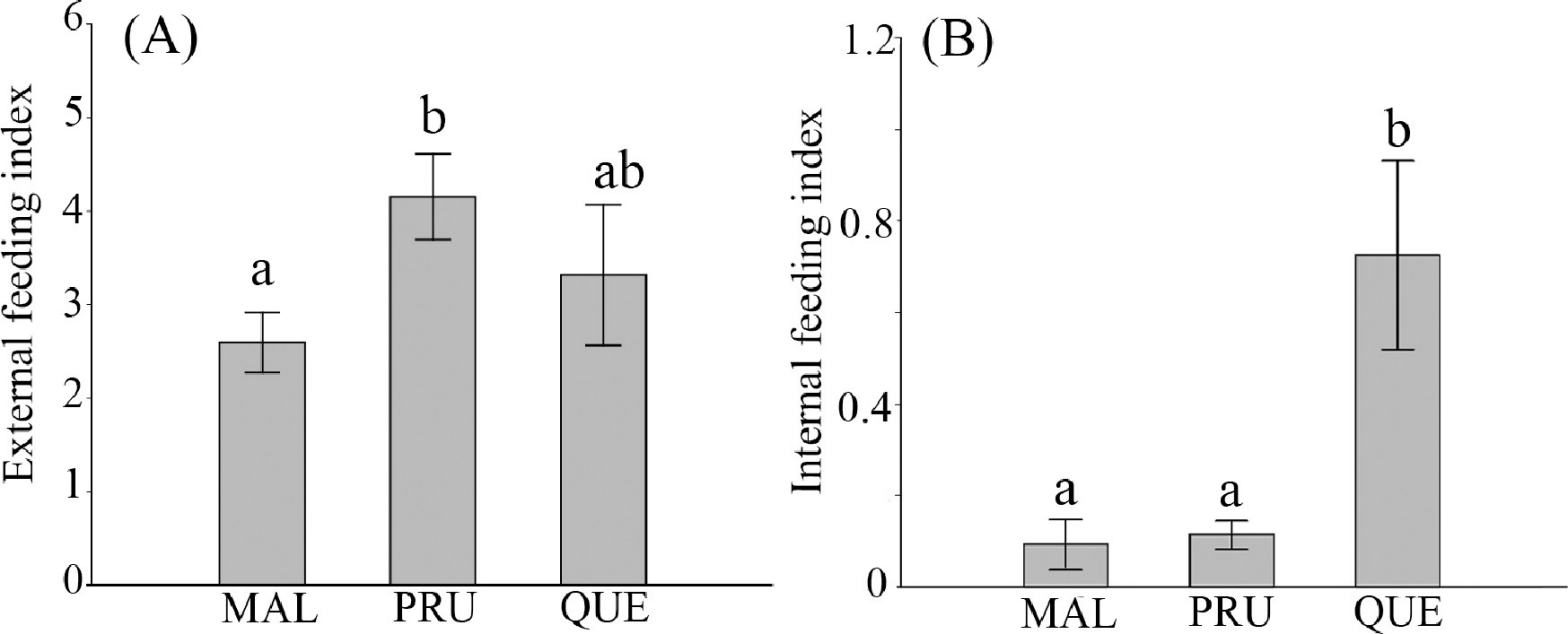
**The chewing rate (A) and the sum of internal feeding (B)(± standard error) of the three plant species across the 18 islands.** Different alphabet above the bar indicates the significant difference at *P* < 0.05.

The average chewing rate varied: PRU 4.15 (±0.46 s.e.)%, QUE 3.32 (±0.75)%, and MAL 2.59 (±0.32)%. The chewing rate of the three plant species across the 18 islands was significantly different (Kruskal–Wallis Chi^2^ = 6.745, *P* < 0.05, Fig 3A), and the chewing rate of the PRU was the highest.

The number of leaves damaged by gallers and miners was 585 (1.90%) and 241 (0.78%), respectively. The number of leaves damaged by gallers was 540 (QUE), 43 (PRU), and 2 (MAL), and that by miners was 133 (MAL), 60 (QUE), and 48 (PRU). The total damage by internal feeders (gallers and miners) was 600 (QUE), 135 (MAL), and 91 (PRU). The internal feeding damages were significantly different among the plants with QUE heavily infested by internal feeders (Kruskal–Wallis Chi^2^ = 23.15, *P* < 0.001, Fig 3B).

There was no correlation between external feeding and internal feeding rates on three plant species even though both phytophagous insects used the same host plant (MAL Pearson r = –0.26 *P* = 0.30; PRU Pearson r = 0.09, *P* = 0.71; QUE, Pearson r = –0.19, *P* = 0.44).

We analyzed the effect of island size and distance from the mainland on phytophagous insect damage. No geographic variable affected external and internal feeders (Table 2). Partitioning of the independent variable to external and internal leaf-feeding guild showed an opposite explanation: distance effect for external and area effect for internal feeding. For food resources, plant species richness was significant to external feeding, while forest cover was to internal feeding guild (Table 3).

**Table 2:** Generalized linear regression for external and internal feeding guilds using geography (a) and habitat diversity and plant resources (b). Std Error, Standard Error, AIC, Akaike Information Criteria. * *P* < 0.0.5.

Dependent variable	Independent variables	Estimate	Std Error	t-value	AIC
(a)					
**External leaf damage**	Intercept	8.38	3.46	2.42*	114.74
	Log (area)	-0.46	2.91	-0.16	
	Log (distance)	5.17	5.89	0.88	
**Internal leaf damage**	Intercept	0.34	0.75	0.46	59.44
	Log (area)	0.64	0.63	1.02	
	Log (distance)	0.39	1.27	0.31	
(b)					
**External leaf damage**	Intercept	0.03	4.87	0.01	110.15
	Elevation	-0.004	0.01	-0.52	
	Plant species	0.02	0.01	2.00	
	Forest cover	0.09	0.05	1.85	
**Internal leaf damage**	Intercept	3.05	1.09	2.81*	56.15
	Elevation	0.00	0.00	-0.52	
	Plant species	0.00	0.00	-0.23	
	Forest cover	-0.03	0.01	-2.35*	

**Table 3:** Summary of hierarchical partitioning for external and internal feeding guilds using geography and habitat diversity and plant resources.

Model	Variables	External				Internal			
		Mal	Pru	Que	Total	Mal	Pru	Que	Total
**Geography**	Area	**52.78**	18.57	6.63	9.41	5	**89.93**	**95.77**	**92.99**
	Distance	47.22	**81.43**	**93.37**	**90.59**	**95**	10.07	4.23	**7.01**
**Food resources**	Elevation	**54.71**	12.58	1.87	3.21	16.61	43.87	2.8	6.84
	Plant species	29.05	**57.33**	**47.26**	53.24*	33.89	3.66	0.06	0.53
	Forest cover	16.21	30.09	50.88	43.55	**49.5**	**52.48**	97.14*	92.63*

Bold indicates the highest explanatory value; an asterisk shows the significant difference at *P* < 0.05 after 999 randomizations.

## Discussion

The island biogeography theory has been previously tested on Korean islands using plants [9], moths [35], and birds [36]. These studies confirmed the dynamic equilibrium model; larger island areas have more species and more distant islands have few species. In contrast, the number of butterflies and staphylinid beetles [6, 11, 14] and insects on Gwanmae-do Island [37] showed that only the island’s area was related to species diversity and that the number of plant species [13] was not affected by island area or distance from the mainland.

Island size is the most informative variable of island biogeography and can be a good surrogate for productivity and food chain length [24, 38]. In this study, the area and distance from the mainland did not affect external and internal feeders on the islands. This result differed from other studies of the effect of island size on consumers [5, 39, 40]. Arnold & Asquith [26] showed a relationship between leaf damage and island size but no relationship with distance. Insect feeding activity was not affected by distance probably because islands offshore of Korea were isolated after the Holocene with no drastic changes in the biology of the island biota [13], and that most islands are close enough to each other to act like stepping stones [6, 11, 41]. Thus, the careful examination of these complex variables should be considered together with the main variables of the dynamic equilibrium theory of island biogeography (area, distance from mainland).

The resource concentration hypothesis explains that high insect density occurs at places with abundant resources, including monocultural areas, high plant densities, and large plant habitats [42, 43]. Since increasing insect density causes more feeding, the examination of leaf damage at high-density areas of insects should show more leaf damage per leaf. In addition, the direct and indirect factors of leaf damage such as vegetation structure, tree age, plant diversity, biomass, leaf physical and leaf traits (LMA, water content, C/N ratio, second metabolites) should be considered when counting leaf damage by phytophagous insects [44–50].

We found that the plant species richness and forest cover played an essential role in the activities of phytophagous insects. However, these two feeding guilds were disproportionally affected by plant diversity and abundance: the external feeder was strongly affected by the number of plant species, while the internal feeder was affected by the forest cover. Leaf damage by chewers differs from galler and miner damage because each feeding guild favors leaves differently. In addition, the diversity of plant species affects the species richness of chewers and miners: chewers favor areas with higher plant diversity. In contrast, miners are negatively affected by higher plant species richness due to the dilution of their preferred host species [51–53]. We found that the leaf density of the three plant groups differed, with MAL the most abundant and the leaf biomass and water content of MAL and PRU being significantly larger than QUE. We predicted that abundant resources, such as MAL and PRU would have greater feeding damage than QUE. Still, this prediction was only partly congruent: the higher external feeding rates in PRU and QUE were not significantly different.

Hiura & Nakamura [32] reported that external and internal herbivores responded differently to leaf traits, including leaf toughness and LMA. Plant with higher leaf toughness and LMA produce thicker leaves, protecting them from external herbivores [32, 54]. In contrast, internal herbivores favor the thick leaves because they can harbor larger herbivores, reduce dryness, and avoid plant chemical attacks by favoring the palisade parenchyma of the leaves [32, 55–57]. Hiura & Nakamura [32] noted that increasing LMA reduced leaf damage by chewers but increased leaf damage by gallers and miners. This study observed that QUE had lighter leaves with little water content and was severely infested by internal feeders. Fernandes & Price [58] suggested that galling insect richness was closely related to hygrothermal harshness; that is, more galling species were found in drier environments. We concluded that leaf traits such as leaf weight, water content, and LMA impacted the leaf damage activities of internal feeders.

We hypothesized that leaf damage would be closely related to island area size and distance, but we found that the leaf damage was not clearly related to island geography, area and distance from the mainland (Table 2). Instead, two feeding guilds were affected differently: external feeder activity was more explained by distance, and internal feeder activity was by area (Table 3). We also hypothesized that the abundance of phytophagous insects was closely related to food abundance. Both plant species richness and forest cover played an important role in phytophagous insects. However, the external feeder was more explained by the species richness, and the forest cover more explained the internal feeder (Table 3). Phytophagous insects attacking three common plants on the Korean islands were differently affected by quantity and diversity of food resources. We conclude that mechanisms affecting phytophagous insects on the Korean islands were linked to amount and different kinds of food resources which indirectly linked to area and distance.

## Supporting information

S1 Data External leaf damage by chewers on 18 islands in southwest Korea. Islands (Is.): 1. Aphaedo, 2. Bigeumdo, 3. Bogildo, 4. Cheongsando, 5. Dochodo, 6. Geomundo (Seodo), 7. Geumodo, 8. Gwanmaedo, 9. Hajodo, 10. Heuksando, 11. Jangsado, 12. Nohwado, 13. Sangjodo, 14. Soando, 15. Wando, 16. Yeondo, 17. Yongsando, 18. Hongdo. Tree species (Tree): 1. *Mallotus japonicas*, 2. *Prunus* spp. 3. *Quercus* spp.
(XLSX)

S2 Data Interrnal leaf damage by gallers and miners on 18 islands in southwest Korea. Islands (Is.): 1. Aphaedo, 2. Bigeumdo, 3. Bogildo, 4. Cheongsando, 5. Dochodo, 6. Geomundo (Seodo), 7. Geumodo, 8. Gwanmaedo, 9. Hajodo, 10. Heuksando, 11. Jangsado, 12. Nohwado, 13. Sangjodo, 14. Soando, 15. Wando, 16. Yeondo, 17. Yongsando, 18. Hongdo. Tree species (Tree): 1. *Mallotus japonicas*, 2. *Prunus* spp. 3. *Quercus* spp.
(XLSX)

S3 Data Leaf traits of three species on 18 islands in southwest Korea. Islands (Is.): 1. Aphaedo, 2. Bigeumdo, 3. Bogildo, 4. Cheongsando, 5. Dochodo, 6. Geomundo (Seodo), 7. Geumodo, 8. Gwanmaedo, 9. Hajodo, 10. Heuksando, 11. Jangsado, 12. Nohwado, 13. Sangjodo, 14. Soando, 15. Wando, 16. Yeondo, 17. Yongsando, 18. Hongdo. Tree species (Tree): 1. *Mallotus japonicas*, 2. *Prunus* spp. 3. *Quercus* spp.
(XLSX)
