# Supplementary material for: The relationship between resource abundance and insect herbivory on islands
Source: PLoS One. 2021 Aug 16;16(8):e0256183. doi: 10.1371/journal.pone.0256183 (PMC8366974; doi:10.1371/journal.pone.0256183)
Supplement: Peer review history

# Peer review: Decision Letter 0

**DOI:** 10.1371/journal.pone.0256183.r001

**Authors:** Kyung-Jin Min

21 May 2021

PONE-D-21-13169

The relationship between resource abundance and insect herbivory on islands

PLOS ONE

Dear Dr. Choi,

Thank you for submitting your manuscript to PLOS ONE. After careful consideration, we feel that it has merit but does not fully meet PLOS ONE’s publication criteria as it currently stands. Therefore, we invite you to submit a revised version of the manuscript that addresses the points raised during the review process.

Please submit your revised manuscript by Jul 05 2021 11:59PM. If you will need more time than this to complete your revisions, please reply to this message or contact the journal office at plosone@plos.org. When you're ready to submit your revision, log on to https://www.editorialmanager.com/pone/ and select the 'Submissions Needing Revision' folder to locate your manuscript file.

Please include the following items when submitting your revised manuscript:

- A rebuttal letter that responds to each point raised by the academic editor and reviewer(s). You should upload this letter as a separate file labeled 'Response to Reviewers'.
- A marked-up copy of your manuscript that highlights changes made to the original version. You should upload this as a separate file labeled 'Revised Manuscript with Track Changes'.
- An unmarked version of your revised paper without tracked changes. You should upload this as a separate file labeled 'Manuscript'.

If you would like to make changes to your financial disclosure, please include your updated statement in your cover letter. Guidelines for resubmitting your figure files are available below the reviewer comments at the end of this letter.

If applicable, we recommend that you deposit your laboratory protocols in protocols.io to enhance the reproducibility of your results. Protocols.io assigns your protocol its own identifier (DOI) so that it can be cited independently in the future. For instructions see: http://journals.plos.org/plosone/s/submission-guidelines#loc-laboratory-protocols. Additionally, PLOS ONE offers an option for publishing peer-reviewed Lab Protocol articles, which describe protocols hosted on protocols.io. Read more information on sharing protocols at https://plos.org/protocols?utm_medium=editorial-email&utm_source=authorletters&utm_campaign=protocols.

We look forward to receiving your revised manuscript.

Kind regards,

Kyung-Jin Min

Academic Editor

PLOS ONE

Journal Requirements:

When submitting your revision, we need you to address these additional requirements.

Please ensure that your manuscript meets PLOS ONE's style requirements, including those for file naming. The PLOS ONE style templates can be found at

https://journals.plos.org/plosone/s/file?id=wjVg/PLOSOne_formatting_sample_main_body.pdf and

https://journals.plos.org/plosone/s/file?id=ba62/PLOSOne_formatting_sample_title_authors_affiliations.pdf

[Note: HTML markup is below. Please do not edit.]

Reviewers' comments:

Reviewer's Responses to Questions

**Comments to the Author**

1. Is the manuscript technically sound, and do the data support the conclusions?

The manuscript must describe a technically sound piece of scientific research with data that supports the conclusions. Experiments must have been conducted rigorously, with appropriate controls, replication, and sample sizes. The conclusions must be drawn appropriately based on the data presented. 

Reviewer #1: Partly

Reviewer #2: Partly

**********

2. Has the statistical analysis been performed appropriately and rigorously? 

Reviewer #1: No

Reviewer #2: No

**********

3. Have the authors made all data underlying the findings in their manuscript fully available?

The PLOS Data policy requires authors to make all data underlying the findings described in their manuscript fully available without restriction, with rare exception (please refer to the Data Availability Statement in the manuscript PDF file). The data should be provided as part of the manuscript or its supporting information, or deposited to a public repository. For example, in addition to summary statistics, the data points behind means, medians and variance measures should be available. If there are restrictions on publicly sharing data—e.g. participant privacy or use of data from a third party—those must be specified.

Reviewer #1: Yes

Reviewer #2: Yes

**********

4. Is the manuscript presented in an intelligible fashion and written in standard English?

PLOS ONE does not copyedit accepted manuscripts, so the language in submitted articles must be clear, correct, and unambiguous. Any typographical or grammatical errors should be corrected at revision, so please note any specific errors here.

Reviewer #1: Yes

Reviewer #2: Yes

**********

5. Review Comments to the Author

Please use the space provided to explain your answers to the questions above. You may also include additional comments for the author, including concerns about dual publication, research ethics, or publication ethics. (Please upload your review as an attachment if it exceeds 20,000 characters)

Reviewer #1: Insect herbivory is an important ecosystem process for nutrient cycling and the rate could be related to the resource diversity and abundance. Authors examine the insect herbivory on 3 plant groups common to the islands from 18 islands in Southwestern part of Korea.

This is fairly well conducted hard work but need sophisticated statistical analysis to make the sound conclusion

Authors had collected ap. 500 leaves from each species and examined the herbivory relative to the feeding types and damage rates. And those data were to relate to the plant characteristics such as resource amount, chemical structures of leaf as well as the island effect. Howver the target of this research seems not clearly defined.

59 About 800,000 insects comprise more than... : Please use the referenced information

83 Previous studies found herbivore-caused leaf damage to be quite rare [24, 25]. ==> this sentence is unclear.

84-88 This part is the main idea and objectives of this paper. However if authors measured the herbivore survival, or even abundance is not clear.

Also leaf chemical traits relative to the herbivory should be introduced.

88-90 Please remove this part and replace relavant to the research hypthesis and title of the manuscript.

In Table 1, Prunus yedoensis was indicated. However in the text, few more species were listed. The same in Quercus group. Please make this clear.

97 maximum height ==> Altitude of the island? Maxium elevation? Please use the term consistantly

106-110 Insect herbivory is differed relative to the season and leaf aging. Please provide information on how you did deal with this problem.

107-108 Sampling plan should be carefully revised. Authors collected leaves from 10 shoots each from 9 plant of each species. And then counted the number of leaves and also measured leaf damages. But authors failed to provide how this information could be related to the resource amoutn in the island.

This was repeated in Line 146 (There was also a significant difference in the total leaves (total number of leaves?) from the plants when we randomly sampled 10 current-year shoots from nine individuals of each plant species). This could be one plant traits but can not related to the resource abundance.

114-119 Leaf damage was averaged out.

120-122 If the number of leaves are different among islands or plant group then average values would be logical for comparision.

124 I wonder if the measured leaf characterisitics could represent the leaf traits of those plants with the sample size of 10 per tree. It is not clear if 100 leaves of each tree or each tree species, and or from each island and so on are not clearly mentioned.

136 I doubt if LMA and water content could be plant chemical characteristics.

149-150 What are the sums of leaf weights is not clear. And why is the sum instead of mean.

148-156 Please separate the intra-specific variability of leaf characteristics and inter-specific variaiton.

166 The chewed proportion from all examined plants : Does this mean the proportion of leaves with chewing damage? Terminology of leaf damage rate, amount and proportion should be used consistant manner. The same for internal feeding damage description.

179 area size ==> area

177-179 How the relationship was signficant should be detailed. If there is a positive relationship betwen a and b and so on.

286 Guildspecific patterns of species richnes ==> Guild specific patterns of species richnes

Reviewer #2: The data are nice but I am not impressed by the analysis. First, I would drop the part about plant leaf parameters. As you have only 3 plant genera in your comparison, you cannot relate herbivory to these parameters in any meaningful way. These plant genera differ with respect to many more parameters which you have not measured (such as those related to secondary compounds) so that relating patterns of herbivory to those few which you have measured (on the basis of a three-point correlation) would necessarily remain speculative. If you will find that such plant data are useful for the Discussion, those may be presented in an appendix but I insist that you refrain from formal analyses. Instead, I strongly suggest that you present a more comprehensive analysis of relationships directly relevant to the island biogeographic framework which still constitutes the core of the paper. I think that a central question of your analysis could be whether the “island effects” (i.e. the effects of area and distance) can be explained by geography as such, or are they mediated by plant communities on the islands. I mean that it is well possible that geography affects insects directly (i.e. they may be less likely to colonise smaller and more distant islands), or such island effect in insects may be a consequence of island effects in their host plants. To answer this question, you should construct models in which both the geographic variables and plant cover variables are included as independent effects, so you can compare their explanatory power. It is great that you have numbers of plant species for each island available (Table 1), may you have access to some other parameters describing plant communities of your islands? You are using distance from the mainland, could you also consider distances between the islands? What about climatic conditions, are they similar enough across the islands to be ignored? At least the smallest islands should be more windy, and less suitable for many insects for this reason? The three groups of tree species should be used in such a way that you primarily ask whether any of the detected patterns are consistent across the three different hosts. Or even more generally, is there a correlation between the rates of herbivory on the three focal hosts across the islands? Similarly, a correlation between the rates of different types of herbivory is of interest. There are a few methodological concerns: it is clear that herbivore damage accumulates throughout the season, so when within the season were your samples taken? Were they taken simultaneously enough on different islands so that phenological differences can be ignored? If not, collection date should be included as a covariate. Your samples were collected in two years, was there an effect of year? You collected shoots of the trees, but the analysis is at the level of individual leaves? If so, “shoot” should be treated as a random variable in the models. Moreover, I would suggest that you consider also the possibility of non-linear relationships. Overall, I think that the results should be better visualized. The map of the area showing the islands would be very helpful, I would also be happy to see graphical presentations of all significant relationships. As I am suggesting major changes in the analysis and overall approach, I currently refrain from detailed comments. In any case, even if am not a native speaker of English myself, I dare to say that your English needs revision.

**********

6. PLOS authors have the option to publish the peer review history of their article (what does this mean?). If published, this will include your full peer review and any attached files.

If you choose “no”, your identity will remain anonymous but your review may still be made public.

**Do you want your identity to be public for this peer review?** For information about this choice, including consent withdrawal, please see our Privacy Policy.

Reviewer #1: No

Reviewer #2: No

[NOTE: If reviewer comments were submitted as an attachment file, they will be attached to this email and accessible via the submission site. Please log into your account, locate the manuscript record, and check for the action link "View Attachments". If this link does not appear, there are no attachment files.]

While revising your submission, please upload your figure files to the Preflight Analysis and Conversion Engine (PACE) digital diagnostic tool, https://pacev2.apexcovantage.com/. PACE helps ensure that figures meet PLOS requirements. To use PACE, you must first register as a user. Registration is free. Then, login and navigate to the UPLOAD tab, where you will find detailed instructions on how to use the tool. If you encounter any issues or have any questions when using PACE, please email PLOS at figures@plos.org. Please note that Supporting Information files do not need this step.

# Author comment: Author response to Decision Letter 0

**DOI:** 10.1371/journal.pone.0256183.r002

6 Jul 2021

5. Review Comments to the Author

Please use the space provided to explain your answers to the questions above. You may also include additional comments for the author, including concerns about dual publication, research ethics, or publication ethics. (Please upload your review as an attachment if it exceeds 20,000 characters)

Reviewer #1: Insect herbivory is an important ecosystem process for nutrient cycling and the rate could be related to the resource diversity and abundance. Authors examine the insect herbivory on 3 plant groups common to the islands from 18 islands in Southwestern part of Korea.

This is fairly well conducted hard work but need sophisticated statistical analysis to make the sound conclusion

Authors had collected ap. 500 leaves from each species and examined the herbivory relative to the feeding types and damage rates. And those data were to relate to the plant characteristics such as resource amount, chemical structures of leaf as well as the island effect. However the target of this research seems not clearly defined.

59 About 800,000 insects comprise more than... : Please use the referenced information

=> We corrected the number and added a reference

83 Previous studies found herbivore-caused leaf damage to be quite rare [24, 25]. ==> this sentence is unclear.

=> We changed the sentence to make clear and deleted a reference.

84-88 This part is the main idea and objectives of this paper. However if authors measured the herbivore survival, or even abundance is not clear. Also leaf chemical traits relative to the herbivory should be introduced.

=> We added the references for supporting our idea and changed the sentence for better understanding.

88-90 Please remove this part and replace relevant to the research hypothesis and title of the manuscript.

=> We added few sentences with references. 

In Table 1, Prunus yedoensis was indicated. However in the text, few more species were listed. The same in Quercus group. Please make this clear.

=> We provided the reason for the grouping of plants in lines 112-114.

97 maximum height ==> Altitude of the island? Maximum elevation? Please use the term consistently

=> We changed this term as maximum elevation throughout the revised ms.

106-110 Insect herbivory is differed relative to the season and leaf aging. Please provide information on how you did deal with this problem.

=> We added the background for this and add a reference.

107-108 Sampling plan should be carefully revised. Authors collected leaves from 10 shoots each from 9 plant of each species. And then counted the number of leaves and also measured leaf damages. But authors failed to provide how this information could be related to the resource amount in the island. This was repeated in Line 146 (There was also a significant difference in the total leaves (total number of leaves?) from the plants when we randomly sampled 10 current-year shoots from nine individuals of each plant species). This could be one plant traits but can not related to the resource abundance.

=> We first sampled the leaves from 10 shoots and the leaves on each shoot differed depending on the plant species. We postulated that even same sampling of each shoot, the plant species have different number of leaves as shown in the results. This different number of leaves could be represented as the resources to feeding insects, even if the number of shoot is the same. 

=> We postulated that if phytophagous insects are abundant, their feeding activity will increase and the evidence of leaf damage will common including these three plant species. There was a pitfall to extrapolate from three plant species into the whole plant community and we provided the reason in the discussion.

114-119 Leaf damage was averaged out.

120-122 If the number of leaves are different among islands or plant group then average values would be logical for comparison.

=> We agreed on your point. However, the number was too small to compare. Therefore we summed in case of internal feeding. We added the sentences to explain this.

124 I wonder if the measured leaf characteristics could represent the leaf traits of those plants with the sample size of 10 per tree. It is not clear if 100 leaves of each tree or each tree species, and or from each island and so on are not clearly mentioned.

=> We first sampled 10 leaves of each tree and grouped these ten leaves as one unit because each leaf was too light to measure. We added an explanation for this.

136 I doubt if LMA and water content could be plant chemical characteristics. 

=> There are many chemical characteristics for plants. Among them, these are included as a characteristic, as we listed in the references. We also changed the word “characteristics” to “traits”.

149-150 What are the sums of leaf weights is not clear. And why is the sum instead of mean.

=> It was mistaken. It was the mean and corrected in the revised ms.

148-156 Please separate the intra-specific variability of leaf characteristics and inter-specific variaiton.

166 The chewed proportion from all examined plants : Does this mean the proportion of leaves with chewing damage? Terminology of leaf damage rate, amount and proportion should be used consistant manner. The same for internal feeding damage description.

=> We tried to use the terminology consistently in the revised ms.

179 area size ==> area

=> We changed into area.

177-179 How the relationship was significant should be detailed. If there is a positive relationship between a and b and so on.

=> We think that the difference between graphs marked with a and b is self-evident as shown in figures. 

286 Guildspecific patterns of species richness ==> Guild specific patterns of species richness

=> We changed into Guild specific patterns of species richness.

Reviewer #2: The data are nice but I am not impressed by the analysis. First, I would drop the part about plant leaf parameters. As you have only 3 plant genera in your comparison, you cannot relate herbivory to these parameters in any meaningful way. 

These plant genera differ with respect to many more parameters which you have not measured (such as those related to secondary compounds) so that relating patterns of herbivory to those few which you have measured (on the basis of a three-point correlation) would necessarily remain speculative. 

If you will find that such plant data are useful for the Discussion, those may be presented in an appendix but I insist that you refrain from formal analyses. 

-=> We agree that the points you commented on the manuscript. However, one of the study aims was to investigate the food resources and herbivore activity, and we believed that some of leaf traits had an important role in the herbivore activity. Thus, we included this part in the revised manuscript, but we deleted the multiple regression between leaf traits and herbivore activity.

Instead, I strongly suggest that you present a more comprehensive analysis of relationships directly relevant to the island biogeographic framework which still constitutes the core of the paper. I think that a central question of your analysis could be whether the “island effects” (i.e. the effects of area and distance) can be explained by geography as such, or are they mediated by plant communities on the islands. I mean that it is well possible that geography affects insects directly (i.e. they may be less likely to colonies smaller and more distant islands), or such island effect in insects may be a consequence of island effects in their host plants. 

To answer this question, you should construct models in which both the geographic variables and plant cover variables are included as independent effects, so you can compare their explanatory power.

=> In the revised manuscript, we analyzed two models by separating biographic framework and food resources. We newly added forest cover data, which showed a strong relationship with area. The total explanatory power was larger when including all independent variables, but we had a problem of multicollinearity. Therefore we tried to analyze two models.

It is great that you have numbers of plant species for each island available (Table 1), may you have access to some other parameters describing plant communities of your islands? 

- We add the forest cover area(%) in the table.

You are using distance from the mainland, could you also consider distances between the islands? 

=> We agree your point. However, the surveyed islands in the present study were relatively large island compared to neighboring island and the distance between islands was generally short. We think this effect is negligible and we discussed the stepping stone effect in the Discussion.

What about climatic conditions, are they similar enough across the islands to be ignored? At least the smallest islands should be more windy, and less suitable for many insects for this reason? 

=> Yes, this could be true. However, unfortunately, the data for the microclimate are largely missing. On the other hand, we observed that most chewers were stick to leaves whenever strong winds and gallers and miners were less affected by windy condition.

The three groups of tree species should be used in such a way that you primarily ask whether any of the detected patterns are consistent across the three different hosts. Or even more generally, is there a correlation between the rates of herbivory on the three focal hosts across the islands? Similarly, a correlation between the rates of different types of herbivory is of interest. 

=> We analyzed the correlation between two types of feeding, but there was no relationsjip across three plant species.

There are a few methodological concerns: it is clear that herbivore damage accumulates throughout the season, so when within the season were your samples taken? 

=> We added the sampling season in the revised ms.

Were they taken simultaneously enough on different islands so that phenological differences can be ignored? If not, collection date should be included as a covariate. Your samples were collected in two years, was there an effect of year? You collected shoots of the trees, but the analysis is at the level of individual leaves? If so, “shoot” should be treated as a random variable in the models. Moreover, I would suggest that you consider also the possibility of non-linear relationships. 

=> We tried to check the effect of sampling using mixed model and provided this result in the revised manuscript: We ran mixed modelling with R (lme function) and found no effect of tree number, shoot number, year and week: the variance of these random variables were 0.1, 9.17*10-7, 9.17*10-7, 3.98% respectively. 

Overall, I think that the results should be better visualized. The map of the area showing the islands would be very helpful, I would also be happy to see graphical presentations of all significant relationships. 

=> We provided the map showing the surveyed islands.

As I am suggesting major changes in the analysis and overall approach, I currently refrain from detailed comments. In any case, even if am not a native speaker of English myself, I dare to say that your English needs revision.

=> Before resubmitting the ms, we again edited the ms through an English editing company.

Attachment Submitted filename: Responses.docx Click here for additional data file.

# Peer review: Decision Letter 1

**DOI:** 10.1371/journal.pone.0256183.r003

**Authors:** Kyung-Jin Min

2 Aug 2021

The relationship between resource abundance and insect herbivory on islands

PONE-D-21-13169R1

Dear Dr. Choi,

We’re pleased to inform you that your manuscript has been judged scientifically suitable for publication and will be formally accepted for publication once it meets all outstanding technical requirements.

Within one week, you’ll receive an e-mail detailing the required amendments. When these have been addressed, you’ll receive a formal acceptance letter and your manuscript will be scheduled for publication.

An invoice for payment will follow shortly after the formal acceptance. To ensure an efficient process, please log into Editorial Manager at http://www.editorialmanager.com/pone/, click the 'Update My Information' link at the top of the page, and double check that your user information is up-to-date. If you have any billing related questions, please contact our Author Billing department directly at authorbilling@plos.org.

If your institution or institutions have a press office, please notify them about your upcoming paper to help maximize its impact. If they’ll be preparing press materials, please inform our press team as soon as possible -- no later than 48 hours after receiving the formal acceptance. Your manuscript will remain under strict press embargo until 2 pm Eastern Time on the date of publication. For more information, please contact onepress@plos.org.

Kind regards,

Kyung-Jin Min

Academic Editor

PLOS ONE

Additional Editor Comments (optional):

Reviewers' comments:

Reviewer's Responses to Questions

**Comments to the Author**

1. If the authors have adequately addressed your comments raised in a previous round of review and you feel that this manuscript is now acceptable for publication, you may indicate that here to bypass the “Comments to the Author” section, enter your conflict of interest statement in the “Confidential to Editor” section, and submit your "Accept" recommendation.

Reviewer #3: All comments have been addressed

**********

2. Is the manuscript technically sound, and do the data support the conclusions?

The manuscript must describe a technically sound piece of scientific research with data that supports the conclusions. Experiments must have been conducted rigorously, with appropriate controls, replication, and sample sizes. The conclusions must be drawn appropriately based on the data presented. 

Reviewer #3: Yes

**********

3. Has the statistical analysis been performed appropriately and rigorously? 

Reviewer #3: Yes

**********

4. Have the authors made all data underlying the findings in their manuscript fully available?

The PLOS Data policy requires authors to make all data underlying the findings described in their manuscript fully available without restriction, with rare exception (please refer to the Data Availability Statement in the manuscript PDF file). The data should be provided as part of the manuscript or its supporting information, or deposited to a public repository. For example, in addition to summary statistics, the data points behind means, medians and variance measures should be available. If there are restrictions on publicly sharing data—e.g. participant privacy or use of data from a third party—those must be specified.

Reviewer #3: Yes

**********

5. Is the manuscript presented in an intelligible fashion and written in standard English?

PLOS ONE does not copyedit accepted manuscripts, so the language in submitted articles must be clear, correct, and unambiguous. Any typographical or grammatical errors should be corrected at revision, so please note any specific errors here.

Reviewer #3: Yes

**********

6. Review Comments to the Author

Please use the space provided to explain your answers to the questions above. You may also include additional comments for the author, including concerns about dual publication, research ethics, or publication ethics. (Please upload your review as an attachment if it exceeds 20,000 characters)

Reviewer #3: The authors satisfactorily addressed most of the concerns, but the manuscript may be published with some revision.

1. In previous review, the authors may present the modified part about reviewer #1 “148-156 Please separate the intra-specific variability of leaf characteristics and inter-specific variation”.

2. L259 area size ==> area

3. Please check

L94: ) -> ]

L162: Figure 1 link error

L202: Table 3 link error

L454: Fig. 3. -> Figure 3

**********

7. PLOS authors have the option to publish the peer review history of their article (what does this mean?). If published, this will include your full peer review and any attached files.

If you choose “no”, your identity will remain anonymous but your review may still be made public.

**Do you want your identity to be public for this peer review?** For information about this choice, including consent withdrawal, please see our Privacy Policy.

Reviewer #3: No

# Editor report: Acceptance letter

**DOI:** 10.1371/journal.pone.0256183.r004

**Authors:** Kyung-Jin Min

6 Aug 2021

PONE-D-21-13169R1 

The relationship between resource abundance and insect herbivory on islands 

Dear Dr. Choi:

I'm pleased to inform you that your manuscript has been deemed suitable for publication in PLOS ONE. Congratulations! Your manuscript is now with our production department. 

If your institution or institutions have a press office, please let them know about your upcoming paper now to help maximize its impact. If they'll be preparing press materials, please inform our press team within the next 48 hours. Your manuscript will remain under strict press embargo until 2 pm Eastern Time on the date of publication. For more information please contact onepress@plos.org.

If we can help with anything else, please email us at plosone@plos.org. 

Thank you for submitting your work to PLOS ONE and supporting open access. 

Kind regards, 

PLOS ONE Editorial Office Staff

on behalf of

Dr Kyung-Jin Min 

Academic Editor

PLOS ONE
